# Real-Time CASSCF (Ehrenfest) Modeling of Electron Dynamics in Organic Semiconductors. Dynamics Reaction Paths Driven by Quantum Coherences. Application to a Radical Organic Semiconductor

**DOI:** 10.1021/acs.jpca.4c06466

**Published:** 2024-11-27

**Authors:** Mercè Deumal, Jordi Ribas-Ariño, Cristina Roncero, Michael A. Robb

**Affiliations:** † Departament de Ciència de Materials i Química Física & IQTCUB, Facultat de Química, Universitat de Barcelona, Martí i Franquès 1, Barcelona E-08820, Spain; ‡ Department of Chemistry, Molecular Sciences Research Hub, Imperial College London, White City Campus 80 Wood Lane, W12 0BZ London, United Kingdom

## Abstract

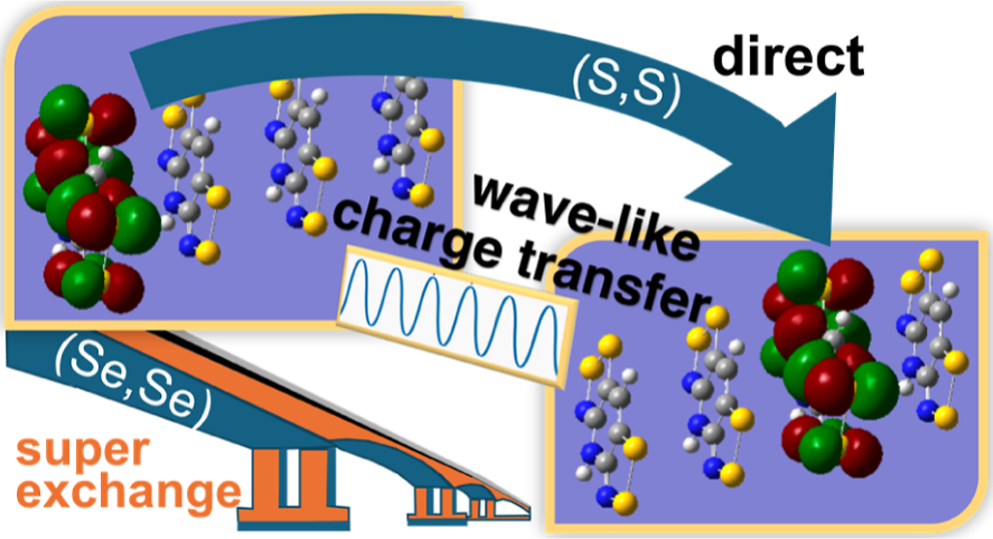

We present a strategy for the modeling of charge carrier dynamics in organic semiconductors using conventional quantum chemistry methods, including the analytic gradient for nuclear motion. The theoretical approach uses real-time CASSCF (Ehrenfest) all-electron dynamics coupled to classical nuclear dynamics for the special case of a small number (4–8) of molecular units. The objective is to obtain mechanistic/atomistic insight at the electronic structure level, relating to spin density dynamics, to the effect of crystal structure (e.g., slippage between spin/charge carriers), and to ferromagnetic and antiferromagnetic effects. The initial conditions for our simulations use the equilibrium structures of all the molecular units. At this geometry, a localized hole on one of the units corresponds to a coherent superposition of adiabatic states. We thus generate a dynamics reaction path driven by quantum coherences. Our aim is to inform experiment and to compare with parametrized theoretical models. The methodology is demonstrated for a perfectly π-stacked ethylene model (up to 8 eclipsed molecular units) for both hole transfer and localized exciton transfer. An application for hole transfer is presented for bisdithiazolyl (S,S) and bisdiselenazolyl (Se,Se) radicals for the special case of ferromagnetic coupling. For these examples, the embedded pyridine radical model organic chromophore (up to 6 eclipsed π-stacked molecular units) has been studied on its own as well as the target bisdithiazolyl (S,S) and bisdiselenazolyl (Se,Se) systems. A significant difference between these systems and the ethylene and pyridine stacks is that the (S,S) and (Se,Se) systems exhibit molecular slippage rather than being perfectly eclipsed. This slippage may result from crystal defects or intermolecular vibrations. For the model systems, the electron dynamics is dominated by the initial and final molecular units, irrespective of the length of the chain. The intervening units act as a “superexchange bridge”. Our simulations reveal that, in the presence of slippage, charge migration cannot propagate across the entire system; instead, the coherence length is limited to 3 molecular units. The results also suggest that the mechanism of charge transport is different for bisdiselenazolyl (Se,Se) (superexchange-like A –[B]→ C) and bisdithiazolyl (S,S) (direct A → C). An analysis of the spin density suggests that, in the charge carrier dynamics, the additional charge carried by the Se versus S in the “scaffold” is small. Since we use a small number of molecular units, the coupled nuclear dynamics is seen to be complementary to the electron dynamics (i.e., creating a hole causes bond length contraction while filling a hole with an electron lengthens the bond). In all the cases studied, the mechanism of charge mobility is wave-like, rather than hopping, because we use the time dependent Schrödinger equation to propagate the electronic wave function.

## 1 Introduction

In recent years, significant efforts have been devoted to the development of theoretical and computational approaches aimed at simulating charge transport in organic semiconductors. All these efforts have contributed to a better understanding of the mechanisms of electron transport in this key class of materials.^1−5^ In this paper we will present a strategy for the modeling of charge carrier dynamics in organic semiconductors using conventional quantum chemistry methods, including the analytic gradient for nuclear motion. As a target example we will look at radical-based^6^ systems, which are very promising compounds for single-component organic conductors and multifunctional materials combining magnetic and conductive properties.^7^ One important feature, which we will document for π-stacked bisdithiazolyl (S,S) and bisdiselenazolyl (Se,Se) radicals,^8^ is that coherence length is limited to 3 molecular units because of the slippage, which occurs in these materials as a result of the crystal packing itself.

There are two different strategies that can be used to study such charge carrier dynamics. One may focus initially on the nuclear dynamics. In this case, the stationary state Schrödinger equation is used to compute the energy and nuclear gradients and, then, one performs nuclear dynamics using surface hopping (see for example^9,10^). Alternatively, one may focus initially on the electron dynamics. Here, one starts with the time-dependent Schrödinger (TDSE) equation and computes the electron dynamics. One then computes the gradient for the nuclear dynamics^11^ (see also exact factorization^12,13^).

The use of the electron dynamics strategy was pioneered by the Heidelberg group.^14^ Initially much of the effort in this field was focused on electron dynamics with fixed nuclei.^15−17^ The reader is referred to the 2015 review by Kuleff and Cederbaum.^18^ The Heidelberg group showed that there is “an intimate relationship between the ionization spectrum of the system and electron dynamics that can be expected after removing an electron from a particular orbital”. Further they speculated on the effect of coupled nuclear motion after very fast movement of the electron from one site to another. Early studies of the effect of nuclear motion on electron dynamics include references to our own work.^19−21^

In a recent work,^22^ we have shown how this electron dynamics approach, starting from the solution of the TDSE, can be used to study electron transfer in a model bis(hydrazine) radical cation. Further, the strategy that involves starting from the TDSE can be used for conventional reaction paths.^23^ Thus, real-time CASSCF nonadiabatic dynamics (second order Ehrenfest) can be used starting from either (1) an adiabatic state progressing through a conical intersection,^23^ (2) a coherent superposition of states, as in attochemistry^24−26^ where the coherent superposition is created with a laser pulse, or (3) as in the case studied in this work, from a coherent superposition of states that results from localization of an electron or hole.^27,28^

Thus, in this work we focus on electron/hole and exciton mobility where the initial conditions involve an electronic configuration with a localized electron/hole or localized exciton electronic state. We choose an initial geometry where the constituent molecule units have their ground state neutral geometry (e.g., from the crystal structure). In our case, the localized hole or exciton state is not an adiabatic state in general. Rather, the creation of the localized state generates superpositions of adiabatic states or coherences that, in turn, drive nuclear motion.^27,28^ This strategy is actually rather similar to a conventional reaction path strategy^29−31^ except that the initial gradient corresponds to the gradient of a superposition of adiabatic states rather than the adiabatic state gradient in the direction of an imaginary vibrational frequency. The analogy fits because the initial geometries of the fragments are all the same: just like a “transition state”. Further, as we shall see, the initial motion involves asymmetric motions within the molecular fragments (i.e., stretching a bond in one fragment and compression in another) much like an asymmetric transition state vector. The alternative strategy, used in the surface hopping approach,^32^ would be to start from an optimized nuclear geometry with one of the units bearing a positive charge. In this case, the initial state would be an adiabatic state.

We now place our work in context of the large number of theoretical approaches that are in use for this type of problem covered in many reviews.^1−5^ In particular, a general surface hopping strategy has been suggested, based on various semiempirical approximations,^32^ such as estimating the coupling matrix elements from fragment overlaps. In contrast, two recent applications^33,34^ have used alternative approaches based on the TDSE, similar to the approach used in this work. Our objective is to model a small number of molecular units with all-electron conventional quantum chemistry. This is thus in contrast to the general surface hopping approach. Our approach is only “unconventional” in that it starts with the TDSE formulation of CASSCF and gradients.^11^ We will limit ourselves to simulations that cover at most a few periods of the electron dynamics because of the computational cost of calculating the analytical gradient. We aim to begin to understand the role of the chemical electronic structure as well as the crystal packing constraints and the nature of the structural changes (e.g., coupled bond length changes) that are directly associated with the electron dynamics. Yet we do not consider the very important effects that result from the coupling of the chromophore to the lattice vibrations of the crystal as well as longer time nuclear dynamics effects. In addition, we will present the results only for a single simulation: effectively, a dynamics reaction path driven by quantum coherences from the TDSE. Most simulation approaches use parametrization methods that are the only way to explore large systems. Thus, the next step would then be to use a sample of initial momenta and treat the nuclei quantum mechanically (we have used the Quantum Ehrenfest, QuEh approach,^35^ in other work). Thus, by focusing in the TDSE and the dynamics driven by the gradient, we avoid the geometry sampling issue that is necessary in surface hopping and other methods^9,10^). As we will demonstrate, the real-time CASSCF (second order Ehrenfest) literally “reveals” the strongly coupled geometric changes from the outset. In our results from a single simulation on a π-stacked ethylene toy model, we will see that the terminal C=C bond length changes are activated by the computed gradients of the TDSE. Alternatively, in a surface hopping and other related approaches, one would “discover” the coupled geometric effects only after the study of many sampled trajectories.

It is important to emphasize that our computations are carried out with no temperature or bath effects (see for example the review of Troisi^5^). Rather, we focus on the initial electronic structure effects only. The problem^36^ of electronic excitation transfer in photosynthesis is very similar and the reader is referred to the review^36^ for additional discussion. The ideas associated with quantum superpositions are familiar from the new field of attochemistry.^37,38^

### 1.1 Theoretical and Methodological Discussion

The main theoretical ideas have been presented in some detail elsewhere.^11^ We use a real-time formulation of the CASSCF method that we shall refer to as the “second order Ehrenfest method”.^11^ In the latter paper,^11^ the gradient computation used the general formulation of Almlöf and Taylor.^39^ In this work, because of the size of the molecules we used a first order approach (no second derivatives) with a smaller step size. We now give the bare essentials (adapted from a recent presentation^23^). The most important point is that we use the full many electron CASSCF wave function with no parameters. The only a priori decision to be made in each case is the choice of active space and the AO basis set. The Ehrenfest method used here has been implemented in a development version of Gaussian^40^ and the 6-31G* AO basis has been used.

In a CASSCF computation, one obtains CI expansion coefficient vectors *C*^*k*^(*t*_*i*_) for each adiabatic state *k* (columns of *U*^*k*^ in eq 2 below) as a function of time, where time is associated with the step number *i* (note we use the notation of Almlöf and Taylor^39^ for the variables). The coefficients *C*^*k*^(*t*_*i*_) are obtained by diagonalization of the CI Hamiltonian. In the real-time CASSCF (Ehrenfest method), rather than use the adiabatic states of the diagonalized Hamiltonian, we introduce a set of coefficients *A*(*t*_*i*_) that mix those adiabatic states and are propagated as the iterative solution of the time dependent Schrödinger equation (TDSE) (see eq 1).
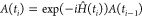
1

We use the time evolution operator (eq 1) to propagate^11^ the solution of the TDSE.
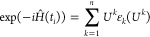
2with
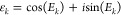
3

The Hamiltonian matrix in eq 1 is the CASSCF CI Hamiltonian and the *E*_*k*_ (eq 3) are the eigenvalues associated with the column eigenvectors *U*^*k*^ (eq 2). The coefficients *A*(*t*_*i*_) then determine the gradients and Hessian (see the discussion of the second order Ehrenfest method in^11^). In this work the coupled nuclear dynamics uses the gradients from the electronic structure part. The gradient driven classical nuclear dynamics per se uses the method of Schlegel et al.^41^ We wish to emphasize that for the computed gradients of the Ehrenfest method, we do not use the average gradient of the states (so-called “mean field” Ehrenfest^33,34^) in the superposition; rather, we use the full expressions for the gradient and Hessian from ref (11). Let us stress that the naïve mean-field approach would involve the average gradient of the two adiabatic states and would not include the cross-interstate mixing terms, which distinguish the superposition from the average. We have discussed the effect of these cross-mixing terms in depth elsewhere.^42−44^

The connection with surface hopping should be mentioned (see ref (45) for a recent discussion). The Ehrenfest method used here, and the surface hopping method are closely related, but only if the nuclear motion in both cases is on adiabatic surfaces. The surface hopping method uses the time dependent Schrödinger equation for electronic motion to decide on a surface hopping probability. However, the nuclear propagation is always on an adiabatic surface. In contrast, in the real-time CASSCF (Ehrenfest) method used in this work, one actually evaluates the gradient of the full Ehrenfest wave function^11^ and uses that to drive nuclear motion. In the present study, the wave function is a coherent superposition of *n* cationic states (where *n* is the number of molecular fragments in the π-stack model). Such a wave function is distinct from its adiabatic components. Further, there is only a single time dependent potential energy surface (PES). Accordingly, concepts associated with surface hopping (e.g., hopping probability between PESs) are not applicable in the present case. However, as we shall show subsequently, with a coherent superposition one may observe that, when the nuclear motion is considered, the population of one adiabatic state may be transferred to another in a surface hopping like fashion.

It remains to re-emphasize the initial conditions in our simulations. We choose an initial geometry where the constituent molecule units have their ground state neutral geometry (e.g., from the experimental crystallographic data). We start with an electronic configuration state function (CSF) with the localized hole or exciton in one of the molecular fragments, which is not an adiabatic state in general but rather superpositions of adiabatic states. These coherences drive nuclear motion.^27,28^ This is the reason why the method used is a type of dynamical reaction path, as discussed above.

## 2 Results and Discussion

### 2.1 Overview

**Figure 1:**
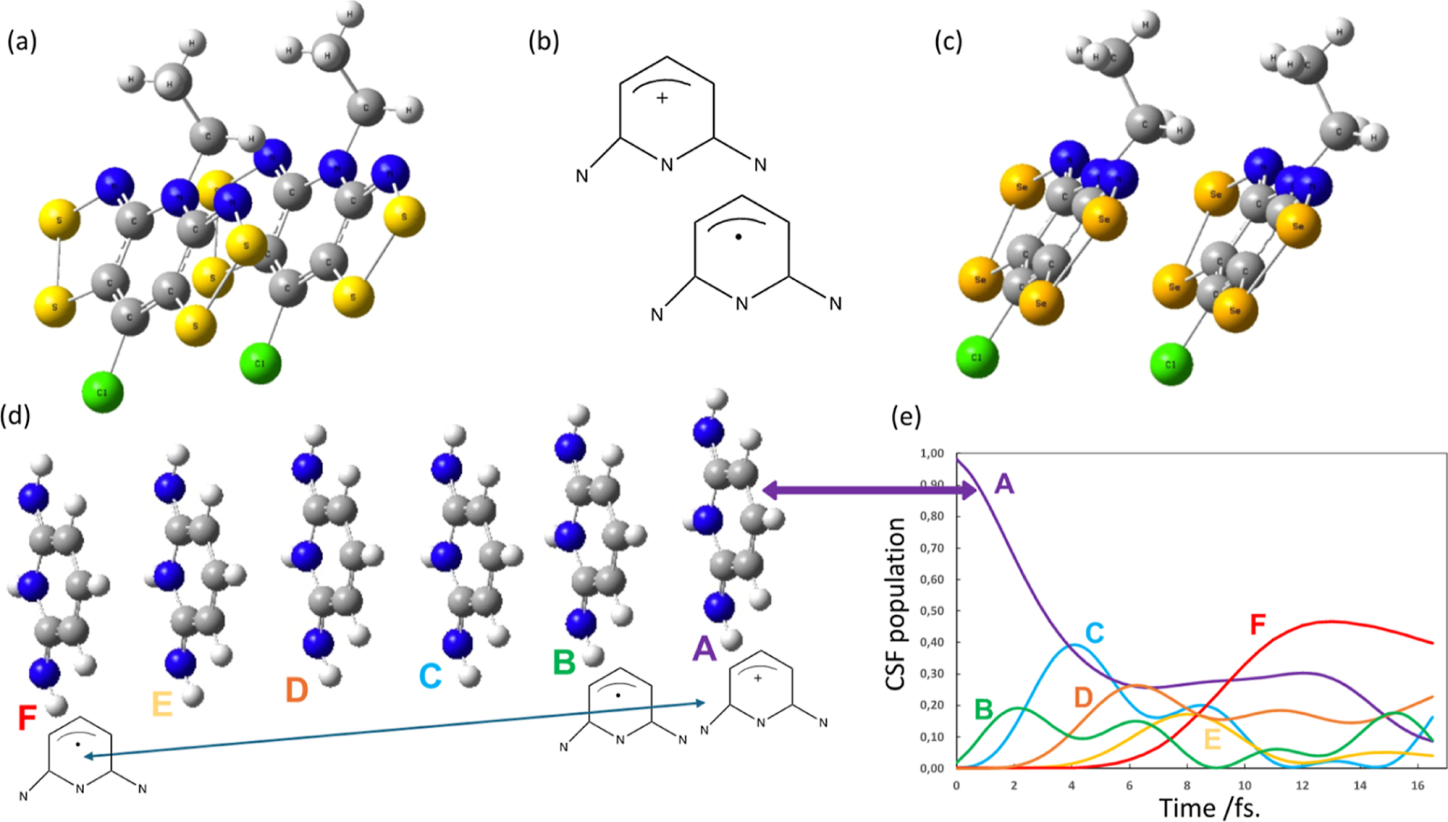
Target organic chromophore radical-based semiconductors showing the “slippage” in the dimer: pyridine-bridged (a) bisdithiazolyl (S,S) and (c) bisdiselenazolyl (Se,Se) compounds.^8^ In our computations Cl atoms and ethylene groups were replaced by hydrogens to produce (b) the model pyridine radical chromophore, where H atoms are not sketched. See in (d) π-stack of 6 perfectly eclipsed molecules of the model chromophore radical cation. (e) Electron dynamics computed for molecules in Figure 1d, where the hole begins on molecular fragment A and moves in a wave-like fashion to molecular fragment F. Note fragments B-E act like a “bridge”.^22^ Note color code associated with each molecular fragment is preserved throughout all figures.

We begin with an overview of some of the ideas that will be developed in this section of the paper, which we have summarized using Figure 1. Our target application is charge transport in two organic radical-based semiconductors, namely a pyridine-bridged bisdithiazolyl-based (S,S) and a pyridine-bridged bisdiselenazolyl-based (Se,Se) materials.^8^ Both radicals form π-stacks in the solid state (see π-dimer excised from the π-stacks of (S,S) and (Se,Se) in Figure 1a,c, respectively, with N in blue, S/Se in yellow/orange, C in gray, Cl in green and H in white). As shown in previous works,^46,47^ the major contributions of the SOMO orbital of these radicals are localized in two allyl moieties of the pyridine chromophore (see Figure 1b). Accordingly, as a reference, we have performed computations of charge carrier dynamics on a π-stack model of 4 and 6 perfectly eclipsed pyridine radicals (see the example with 6 molecular units in Figure 1d).

The analysis of the resultant electron dynamics for a model with 6 eclipsed pyridine chromophore units shows the computed weights of the configuration state functions (CSF) of the time-dependent wave function as a function of time (see Figure 1e). The active orbitals in each unit are localized on each fragment (A to F). Each CSF has a hole on one site/fragment (i.e., the active orbital is empty) from molecular fragments A to F with the remaining active electrons all spin parallel (i.e., the multiplicity in this case is 2S + 1 = 6). Therefore, this is a ferromagnetic case. One can see that the hole moves from A to F in 12 fs and that the intermediate molecular units B–E act like a superexchange bridge (see Figure 1e). As we will discuss subsequently, this dynamics is only slightly perturbed by the synergistic, coupled, nuclear motion which is not included in Figure 1e.

### 2.2 Illustration of Hole and Exciton Propagation in Perfectly Eclipsed π-Stacked Ethylenes

**Figure 2:**
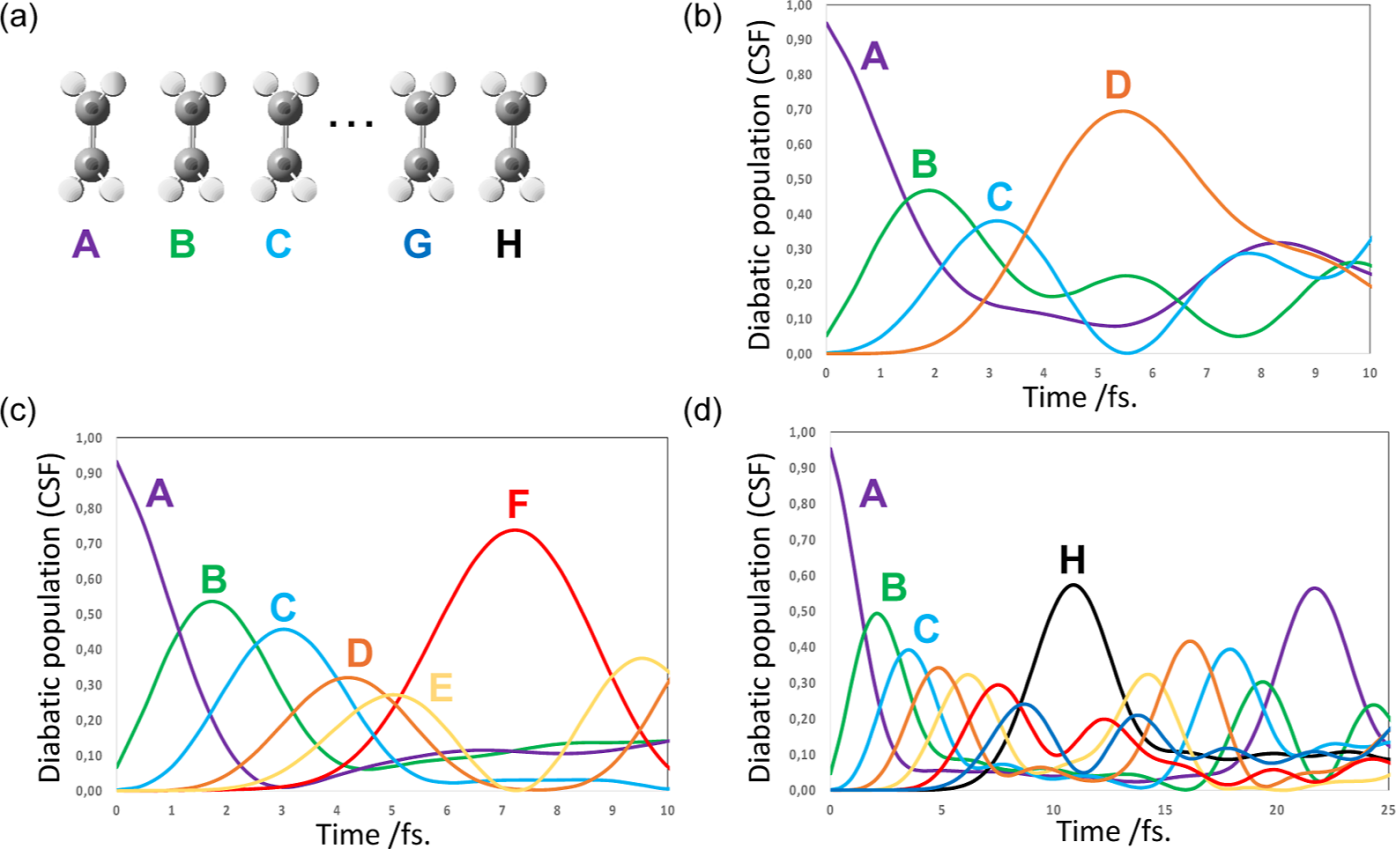
(a) Toy models composed of perfectly eclipsed π-stacked ethylenes containing from 4 to 8 units (A to H fragments). Hole propagation for (b) 4, (c) 6, and (d) 8 ethylene units (fixed nuclei). The labels A, B,.. etc. correspond to the weight of the CSF where the hole is located. In all plots, the purple curve corresponds to the CSF A where the hole is localized on unit A, the green curve to CSF B where hole is on unit B, etc. Note that there is a color code associated with each molecular fragment which is preserved throughout all figures. In parts (b) and (c), we show only the propagation for the first complete transfer. For part (d), we only show a complete period of CSF A (ca. 22.5 fs).

In order to establish some general ideas, we now give a demonstration of hole and exciton migration in a toy system of π-stacked ethylenes containing 4, 6, and 8 units perfectly eclipsed (see Figure 2a) with an equal spacing of 4 Å. Note this same model example has been previously used in a surface hopping algorithm,^1,2^ with the usual momentum and position sampling. The hole migration was simulated with a CAS (2*n*-1,*n*) doublet where the *n* active π orbitals were localized on the *n* individual ethylene units. For the exciton case, we used an orbital basis of π and π* on each ethylene obtained as Natural Bond Orbitals NBO^48^ from the CAS (2*n*,2*n*) where *n* is again the number of ethylene units. Note that the active π orbitals for the “real” excited state of ethylene would involve diffuse Rydberg-like functions. Yet, this is a “toy” example in which this effect is neglected. In each case, we started from a fully converged CASSCF computation where the orbitals were transformed either to localized orbitals (hole migration) or to NBO (exciton migration) before the Ehrenfest computation was carried out.

**Figure 3:**
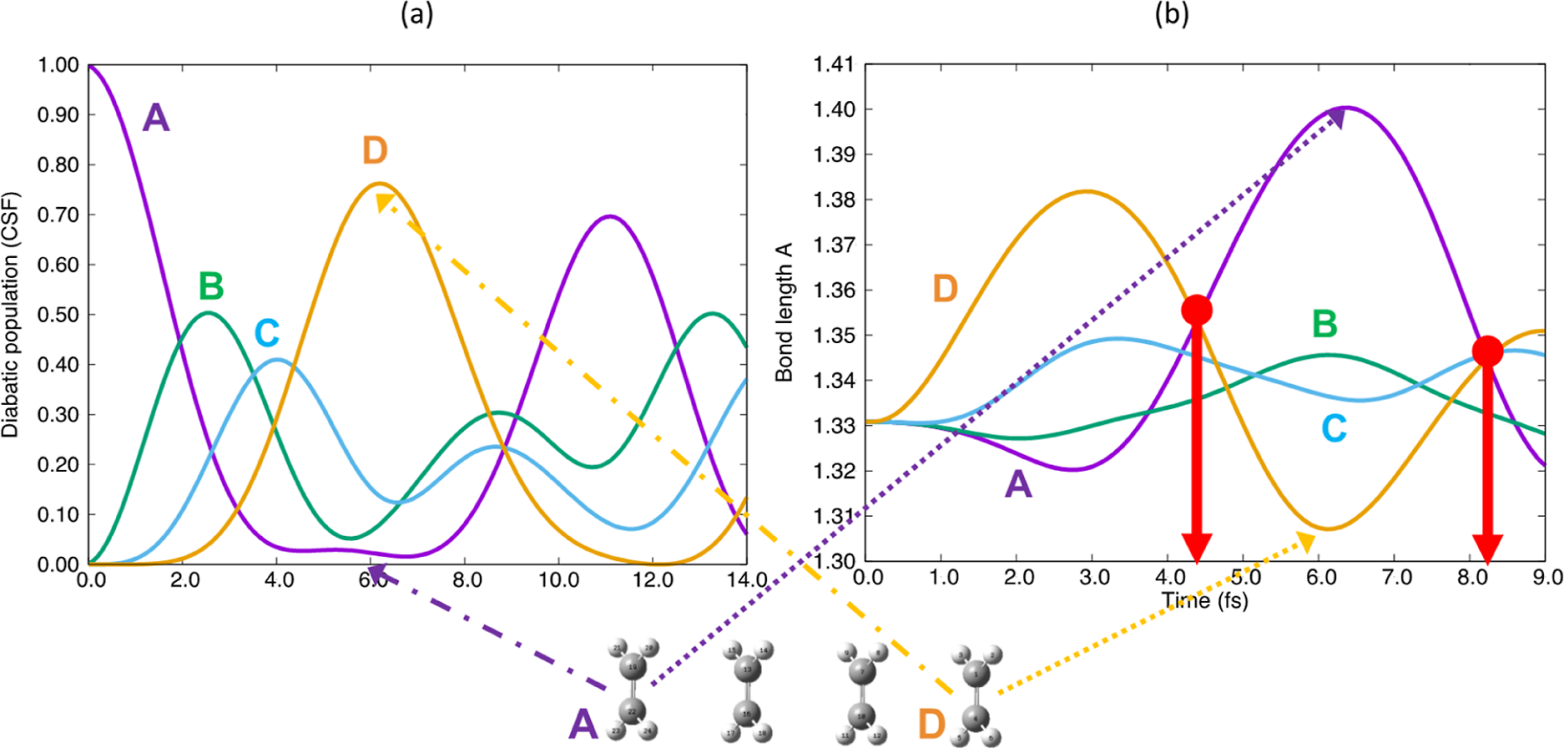
Charge migration in a perfectly eclipsed π-stack of 4 ethylene units with nuclear motion: (a) CSF weights and (b) CH_2_=CH_2_ bond lengths. The red bar in (b) indicates “a transition state like” geometry where the A and D bond lengths are equal. Note that the time scale used in part (a) is longer than in Figure 2b because we show a full period of CSF A and in part (b) we then zoom in to highlight the main changes in bond lengths.

**Figure 4:**
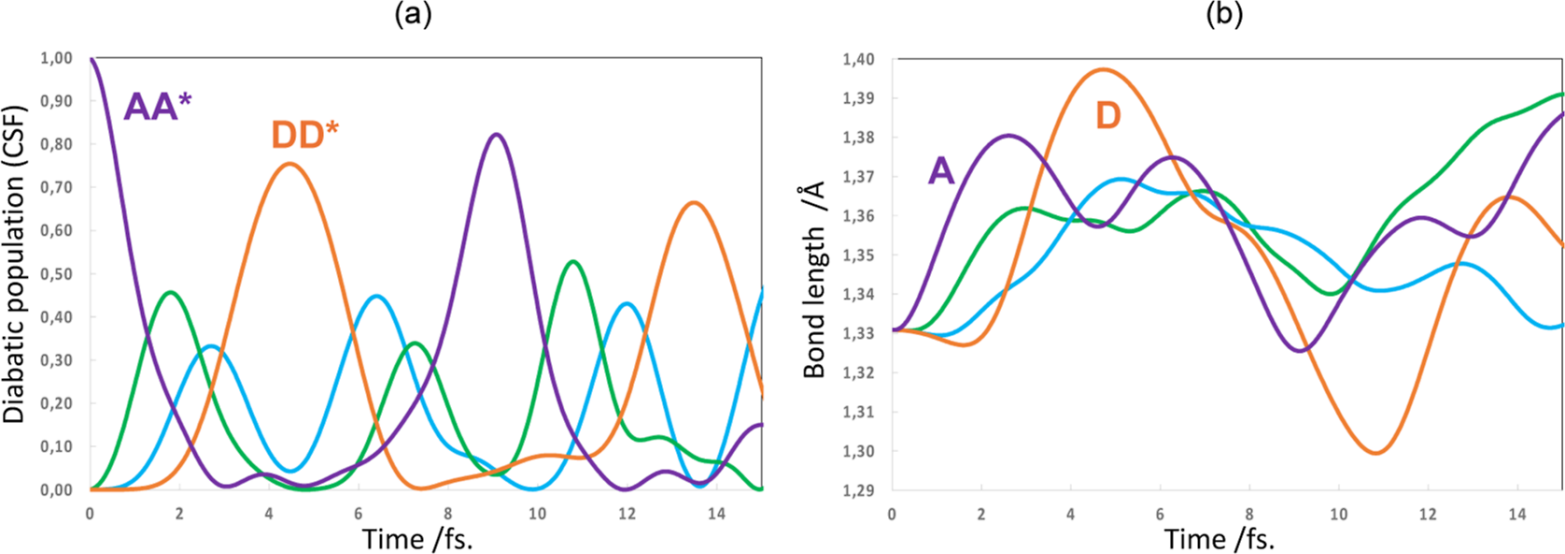
Exciton migration with coupled nuclear motion in a perfectly eclipsed π-stack of 4 ethylene units: (a) CSF population and (b) CH_2_=CH_2_ bond lengths. Note that the time scale used in part (a) is the same as in Figure 3a for comparison purposes between charge and exciton migration.

Figure 2 gives the results for hole migration with frozen nuclei for the 4, 6, and 8 π-stacked ethylene examples (see Figure 2a for a schematic representation of the geometries.). Then, following on, Figure 3 and 4 give the corresponding results for a 4-molecule ethylene unit for both hole and for exciton migration but with the coupled nuclear motion included. Thus, Figure 3a/b correspond to hole migration in a 4-ethylenes perfectly eclipsed π-stack with gradient driven nuclear motion and Figure 4a/b correspond to exciton migration also with gradient switched on. Note that the time axis is slightly different in these figures because the period of electron and nuclear dynamics changes. These effects have been noted in the captions.

In Figure 2, one can see that there is charge migration from the initial molecular unit A to the final one (namely, D, F and H in 2b, 2c and 2d, respectively), via a superexchange bridge involving transient population of the intervening molecular sites. Thus, starting from the coherent superposition of the adiabatic states, we see wave-like transport with a *ℏ*/Δ*E* period similar to that postulated in biological systems.^36^ These results are similar irrespective of the number of molecular units being 4, 6, or 8. Thus, for more complex systems as our (S,S) and (Se,Se) target systems, it seems one may get mechanistic information with just 4 units.

Now let us turn our attention to the coupled nuclear motion. We refer to Figure 3a,b for hole and Figure 4a,b for exciton transport. Notice that with coupled nuclear motion the CSF populations for both hole transport (Figure 3a) and exciton transport (Figure 4a) show a behavior that is similar to the hole transport shown with frozen nuclei (Figure 2b). Only the period and amplitude of the oscillations changes with coupled nuclear motion. The bond length changes are complementary to the CSF changes and involve out of phase oscillations of the A and D bond lengths in synch with the corresponding wave function oscillation. Thus, in Figures 3b and 4b we have asymmetric out of phase molecular motion (between A and D: when *d*_A_ shortens, *d*_D_ enlarges and vice versa) that is effectively like a chemical reaction path driven by the oscillation of the wave function. Of course, there will be dephasing or loss of coherence at longer times and with the inclusion of lattice vibrations. Let us remark that the results for both hole and exciton propagation are similar.

Finally, let us comment briefly on the coherent superposition itself corresponding to the initial condition with a hole localized on CSF A. Here the results are subtlety different from surface hopping. The adiabatic state coefficients, i.e., the rows in Table 1, correspond to the linear combinations of CSFs to construct the adiabatic states for the π-stack of 4 eclipsed ethylenes, *U*^*k*=1–4^. These states are completely delocalized as required by symmetry. The columns in Table 1 correspond to the linear combination of adiabatic states corresponding to a localized hole. Thus, the initial superposition with the hole on site A (labeled CSF A) is just the first column of Table 1. Note that the initial state with the hole localized on site A was constructed from an appropriate coherent superposition of the four adiabatic states.

**Table 1:** Coefficients of the Adiabatic State are Given as Rows in Terms of CSF A–D^a^

superposition corresponding to a localized hole in fragment					
adiabatic state *k*	(*E*_*k*_ – *E*_1_)/eV	A	B	C	D
*U*^*k*=1^	0.0000	+0.3	–0.6	+0.6	–0.3
*U*^*k*=2^	0.4462	+0.5	–0.5	–0.5	+0.5
*U*^*k*=3^	0.8308	–0.6	–0.3	+0.3	+0.6
*U*^*k*=4^	1.0213	+0.5	+0.5	+0.5	+0.5

a The CI vectors (*U*^*k*=1-4^) of a 4-unit ethylene π-stack are linear combinations of CSF with a hole on A, B, ... etc. (in each case the numbers have been rounded up to 1 decimal point for clarity). Each column (A to D) corresponds to the initial superposition of adiabatic states corresponding to localization of hole on A, etc. at initial time (*t* = 0 fs).

**Figure 5:**
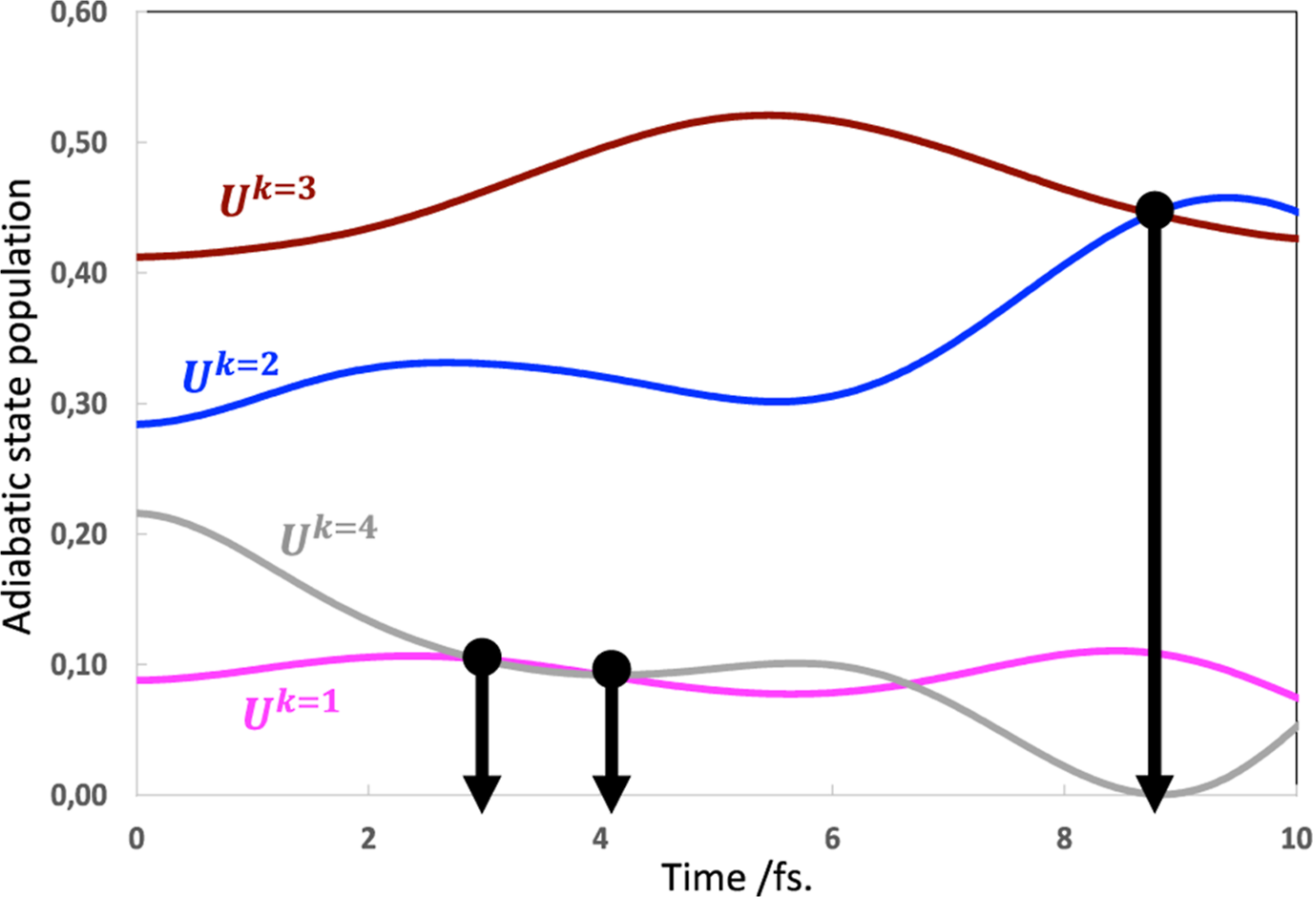
Adiabatic state *U*^*k*=1–4^ populations for a π-stack of 4 eclipsed ethylene units (corresponding to Figure 3). The vertical bars emphasize the adiabatic state crossings. Note that the time scale is the same as Figure 3b to emphasize that the adiabatic states cross on different time scales.

Now we can make a connection with surface hopping methods (see reviews^1,2^). To begin with, our initial geometry has all the ethylene units at their ground state neutral geometries (like a transition state for hole migration), whereas in a surface hopping approach one starts with one ethylene unit at a cation optimized geometry. In Figure 5, we show the adiabatic state populations as a function of time corresponding to Figures 3a,b (*t* = 0 corresponds to data of Table 1). We have emphasized the regions where the populations of any two distinct states become equal with vertical lines. Notice that adiabatic states 1 and 4 cross around 3–4 fs, while 2 and 3 cross at ca. 8.5–9.0 fs (note that both cases are same as diabatic CSF A and D in Figure 3a). Remarkably, these correspond to points where the A and D bond lengths are approximately equal (see Figure 3b). Such points would be where diabatic states A and D cross. Note, however, that the population crossings occur between states 1/4 and 2/3 rather than between adjacent states as in surface hopping. Thus, we observe population transfers between adiabatic states as in surface hopping; however, the population transfers occur between states that are not degenerate (e.g., states *k* = 1 and 4, see Table 1). Since, the bond lengths of A and D are indeed equal (as would happen in a transition state and the energy of the diabatic states would be equal), the real-time CASSCF can be thought of as a more general form of multidimensional surface hopping approach, except that there is only one time-dependent potential surface (corresponding in the toy model of a π-stack of 4 perfectly eclipsed ethylenes to the superposition of 4 cationic states).

Let us briefly summarize the main conclusions from the demonstration using perfectly eclipsed π-stacked ethylenes. We expect that some generalizations can now be made, tentatively. First, the exciton and hole transport when nuclear motion is included (Figures 3a/b and 4a/b, respectively) show that the nuclear motion is in synchronization with the electron dynamics and that the motion is dominated by the first and last molecular unit. We have noticed such synchronization in previous hexatriene cation simulations.^27^ From Figure 2, we can see that the electron dynamics is broadly similar for 4, 6, and 8 units. Therefore, in large π-systems one may get useful information with a simple 4-unit model. From Figure 5, it seems that one has population transfer between adiabatic states as in surface hopping; this is indeed associated with geometry changes, but it is not induced by a crossing of adiabatic states. Further, the geometry change is derived from the electron dynamics rather than an adiabatic state change (hop between surfaces).

### 2.3 Application to Organic Radical-Based Semiconductors: Pyridine-Bridged Bisdithiazolyl (S,S) and Bisdiselenazolyl (Se,Se) Compounds

**Scheme 1:**
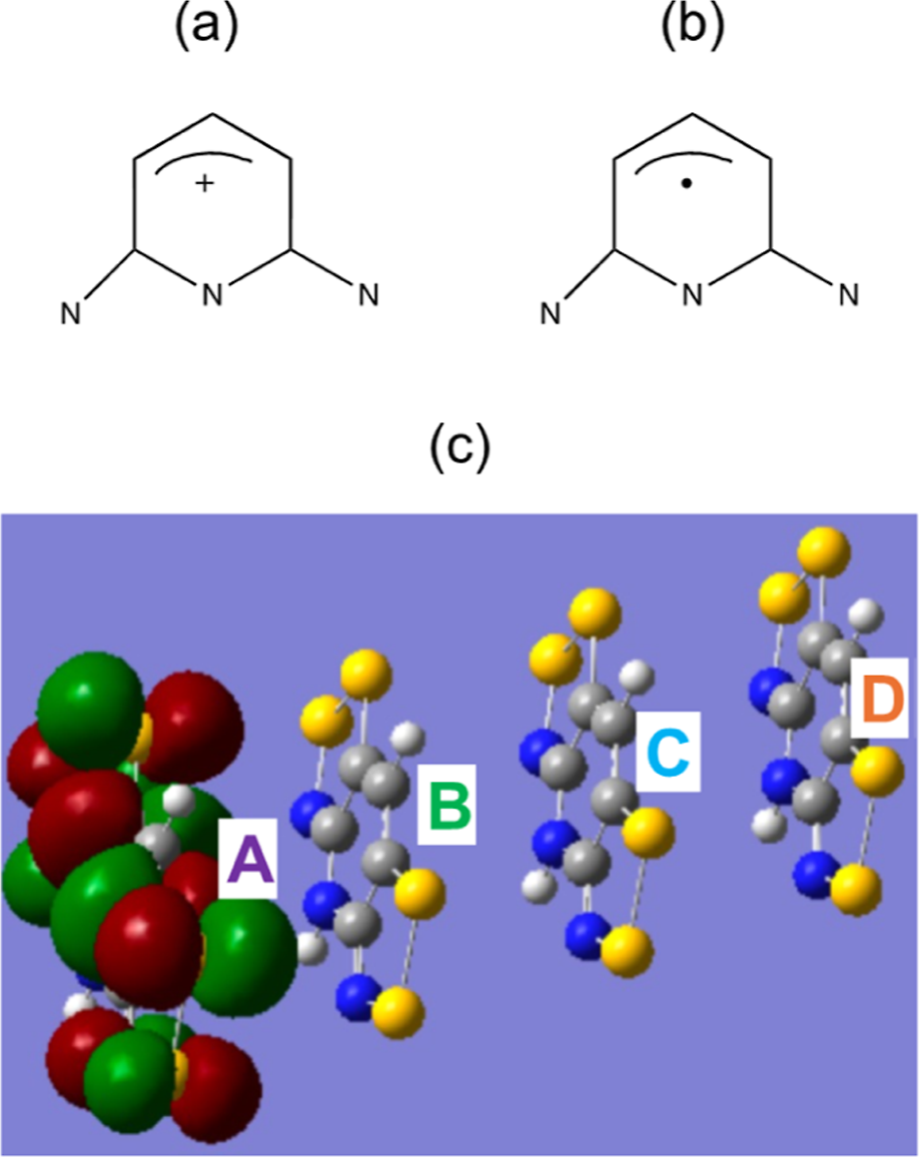
Bisdithiazolyl (S,S) Cationic and Neutral VB Electronic Structures of a Pyridine Chromophore Unit and Active Orbital Centred on Fragment A of Bisdithiazolyl (S,S)

We will now discuss the application of these ideas to two well-characterized organic radical-based semiconductors, namely, pyridine-bridged bisdithiazolyl (S,S) and bisdiselenazolyl (Se,Se) compounds^8^ (see Figures 1a,c, respectively). They belong to one of the most important families of single-component radical multifunctional materials.^8,49−72^ To get more insight into the charge transfer mechanism, we also consider a model of perfectly eclipsed π-stacked pyridine radical chromophores (with 4/6 molecular units, see Figure 1d for an example) to assess the effect of heteroatoms (S and Se) in the electron dynamics. As a reference, we will here consider 4 molecular unit models since we will use 4 molecular units in the target (S,S) and (Se,Se) systems. Note that in all our computations we use an active space of localized MO (see an example of MO associated with CSF A for bisdithiazolyl (S,S) in Scheme 1c, where initially this orbital is empty (hole) and corresponds to the cationic VB structure represented in Scheme 1a. As the hole moves to B or C, this orbital becomes occupied, corresponding to the neutral VB structure in Scheme 1b ).

In radical organic semiconductors, a new feature arises that is characteristic of a system built from radicals: namely, several spin multiplicities are possible (see the book of McWeeny^73^ for an introductory discussion of spin coupling). For instance, for a 6 radical system with 5 electrons, spin multiplicity (2S + 1) will encompass values from 6 (highest spin state where all radical spins align parallel) to 2 (lowest spin state with maximum number of radical spins coupled). We will hereafter discuss the case of model pyridine radical chromophores with highest spin multiplicity, i.e., the ferromagnetic case. These computations were carried out in exactly the same fashion as the π-stacked ethylene but with a different active space since we deal with a stack of radicals (e.g., a 6-unit system with 5 electrons required a CASSCF(5,6) with 2S + 1 = 6). Let us mention that we have also carried out simulations with a lower spin multiplicity (e.g., 2S + 1 = 4), which result in both hole migration and spin exchange between the sites. In this case, the computations for the perfectly eclipsed 6-unit pyridine system were carried out with an initial superposition of CSF’s corresponding to state A that weighted the 4 spin eigenfunctions for CSF A equally. The subsequent analysis also averaged of the 4 spin eigenfunctions for each diabatic state B, C···F. The results were almost identical to the high spin ferromagnetic case and will not be discussed. However, this is a subject for future detailed investigation.

**Figure 6:**
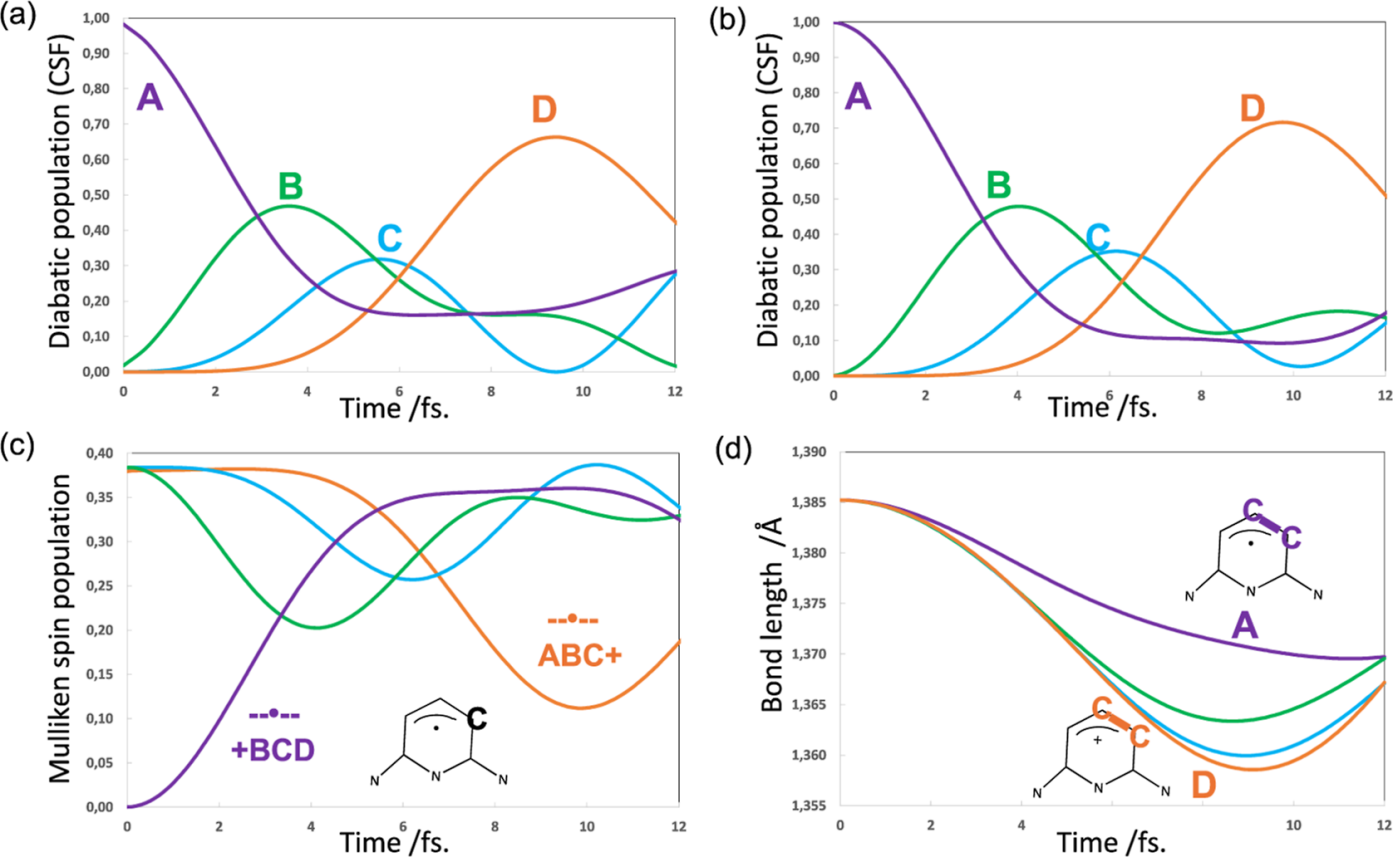
Results for the perfectly eclipsed π-stack of 4 model pyridine radical chromophore system: (a) CSF population with frozen nuclei; (b) CSF population with coupled nuclear motion; (c) spin density on the terminal allyl carbon atom with frozen nuclei; and (d) C–C bond length in the C–C–C allyl fragment with coupled nuclear motion. See atoms used in (c,d) schematized inset.

Given that we will use 4 molecular units in the target (S,S) and (Se,Se) systems, we shall consider the results including the nuclear motion for the model pyridine radical chromophore with 4 molecular units as a reference. Results summarized in Figure 6a,b show the CSF weights with nuclear motion off and on, respectively, which in this short simulation are quite similar. We will thus continue analyzing the data at the atomistic level with nuclear motion off.

To analyze the results at the atomistic level, we will use the spin density^74^ (see Figure 6c for spin density on one of the terminal carbon atoms of the allyl radical fragment of the model pyridine radical chromophore). In the case of the spin density, the hole is located in the fragment whose spin density approaches zero. One can indeed see that around 10 fs. The spin density on fragment D goes to a minimum (∼0.10) at the same time as the population on site D goes to a maximum (∼0.70) (see Figure 6a,b). In computations on the target system, the S and Se atoms are also involved as charge carriers as well as the organic chromophore (see Figure 1a,c, respectively), and the spin density can be used to distinguish the S and Se atoms contributions, as we shall discuss presently. Finally, in Figure 6d, we plot the C–C bond length for one of the C–C bonds in the C–C–C allyl fragment (Scheme 1a,b). One can again observe the synergistic nature of the C–C bond length variation with the change from CSF A to D, i.e., the allyl C–C bond D gets shorter as the hole migrates to D (∼10 fs.).

Referring still to the perfectly eclipsed model pyridine radical chromophore and focusing on the larger 6-unit example in Figure 1d,e, we would like to point out the remarkable similarity of the population between the model with 6 pyridine-radical chromophores (Figure 1e) and the 6-unit ethylene π-stack (Figure 2b): the overall charge migration takes place from A to F and uses the intermediate sites as a bridge. Thus, once again, results for the larger 6-unit model are consistent with conclusions drawn from the smaller 4-unit model.

Let us turn now to the (S,S) bisdithiazolyl and (Se,Se) bisdiselenazolyl target systems (see Figure 1a,c, respectively). The computations were carried out in exactly the same fashion as for the perfectly eclipsed model pyridine radical chromophore. We used a tetramer of either (S,S) or (Se,Se) moieties with the Cl atoms and the ethylene fragment replaced by H atoms in the interests of computational costs. The major differences in the target molecules vs ethylene or model pyridine systems are (1) the singly occupied orbitals are fully delocalized over each fragment as shown in Scheme 1c (i.e., on the S or Se atoms) but remain localized on fragment sites, and (2) the molecular units are “slipped” with respect to their adjacent upper and lower units (see Figure 1a,c).

**Figure 7:**
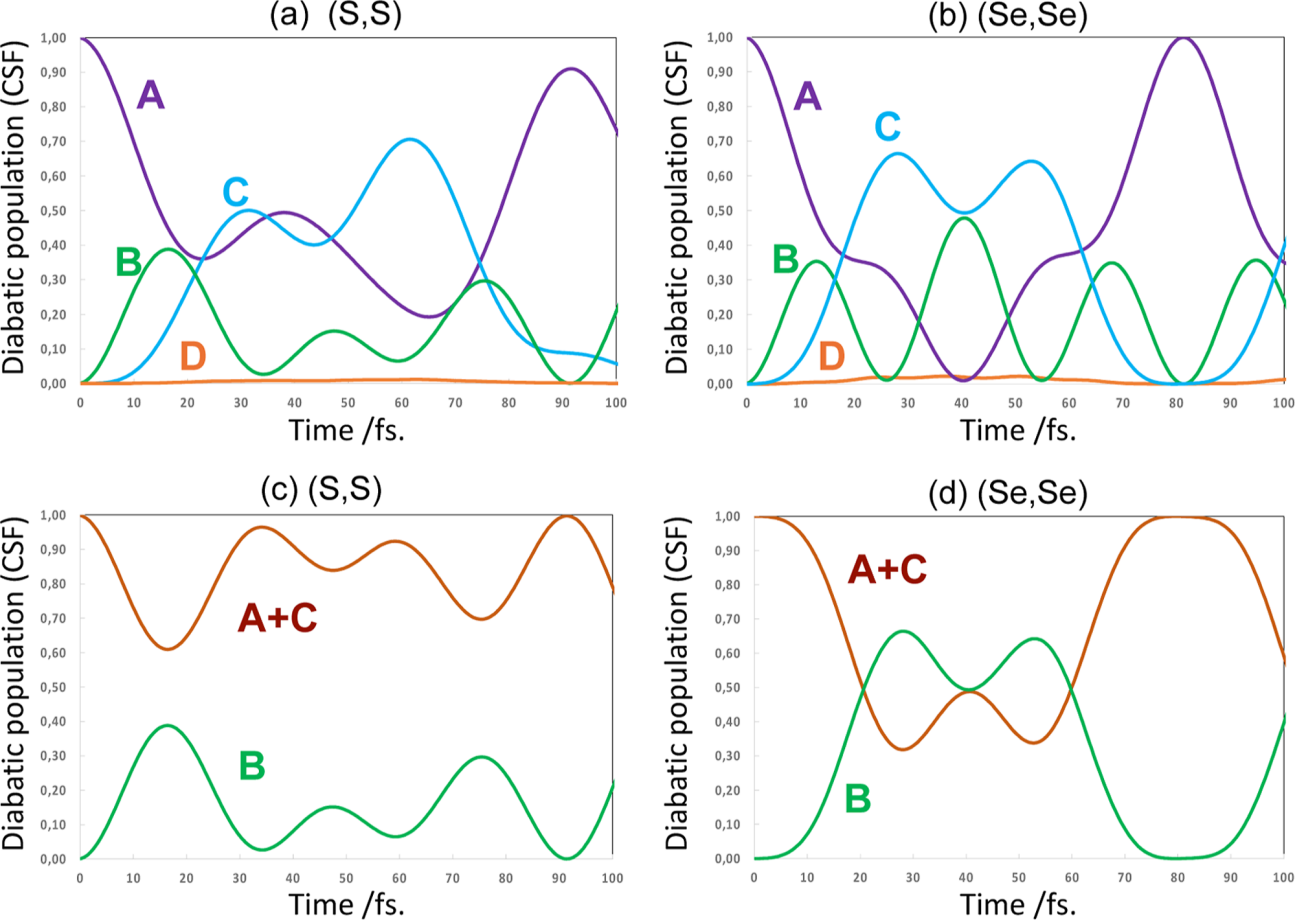
Individual CSF A-D populations using a tetramer A–D model of (a) (S,S) bisdithiazolyl and (b) (Se,Se) bisdiselenazolyl radicals. Note that CSF D has zero population in both cases. CSF A + C vs B populations for (c) (S,S) (note A → C direct coupling with little contribution from B) and (d) (Se,Se) (note A and C are strongly coupled with B as bridge resulting in A –[B]→ C superexchange coupling). The model used is a tetramer A–D model with Cl atoms and ethylene fragment replaced by H atoms.

Computed CSF populations for bisdithiazolyl (S,S) or bisdiselenazolyl (Se,Se) 4-unit system show that we get ultimate population transfer between A and C in both cases (see Figure 7a,b). Interestingly, it turns out that D is not populated because of the slippage, which results in charge migration not propagating across the entire system. Thus, the coherence length is 3 rather than being infinite as in the perfectly eclipsed both π-stacked ethylene and π-stacked model pyridine radical chromophore. This coherence length might be aligned with the polaron length.^5^ In this case the coherence length is smaller because the slippage results in a smaller overlap between the more distant A-D localized states.

In contrast to the perfectly eclipsed model pyridine system, the electron dynamics for (S,S) versus (Se,Se) is quite different. This difference is mainly due to the different involvement of the bridge unit B (see Figure 7c,d, where we show the sum of A + C vs B diabatic populations). In (Se,Se) bisdiselenazolyl, we have an A to B to C sequential transfer or bridge superexchange (see Figure 7d), more like the eclipsed both model pyridine radical chromophore and the ethylene π-stack. In (S,S) bisdithiazolyl, the bridge unit B is only weakly involved, and we have more or less direct A → C transfer (see Figure 7c). Thus, in the end, the bisdithiazolyl and bisdiselenazolyl charge carrier mechanisms are different. In recent work^22^ involving 2 chromophores (A and C) with a linker B, we were able to demonstrate both superexchange (A –[B]→ C) and direct (A → C) charge transfer depending on the initial superposition of four cationic states. Here, initial superposition of 4 (S,S)/(Se,Se) cationic states is the same. Yet, the outcome points toward two different charge transfer mechanisms.

**Figure 8:**
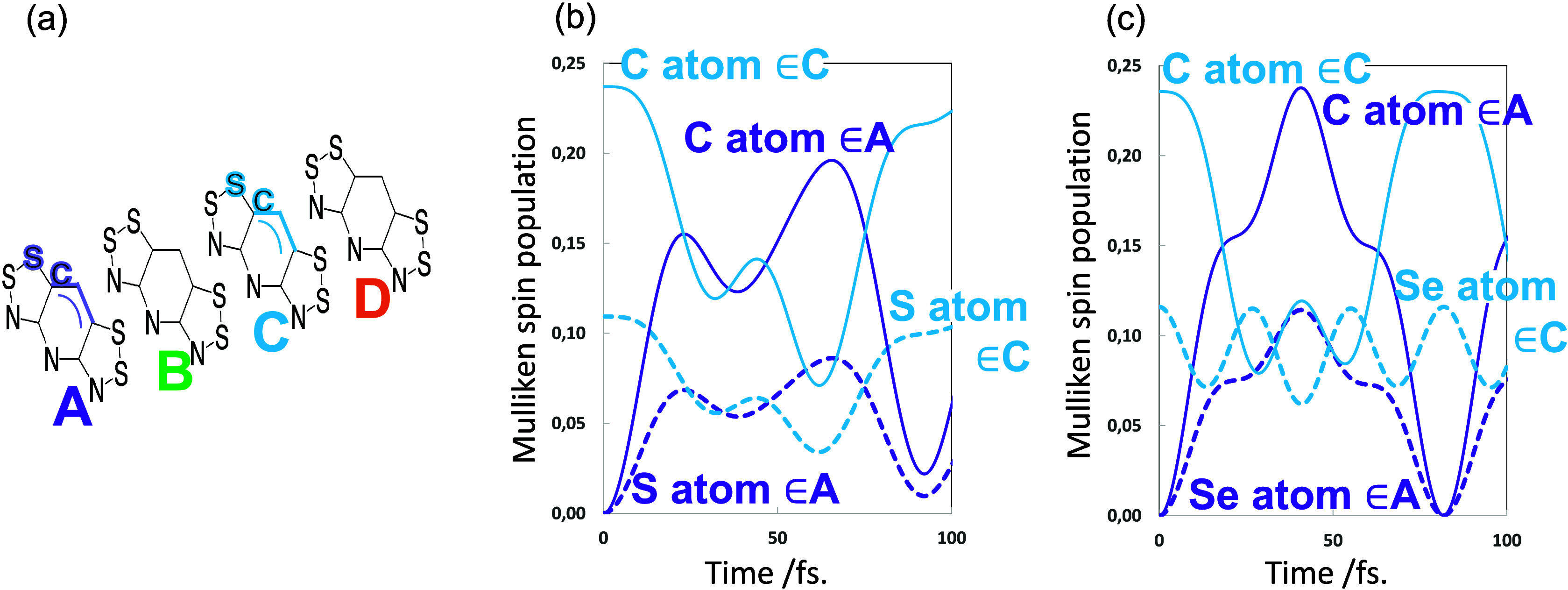
(a) Tetramer A–D model with Cl atoms and ethylene fragment of (S,S) bisdithiazolyl replaced by H atoms. Same model but changing S by Se applies for (Se,Se) bisdiselenazolyl. Spin densities on allyl C atom and S/Se atoms of A and C molecular units highlighted in (a) for (b) (S,S) bisdithiazolyl and (c) (Se,Se) bisdiselenazolyl.

In Figure 8 we show the spin density as a function of time for one of the allyl terminal carbons and for the S and Se atoms bound to the allyl fragment. They have the same oscillatory behaviors as the CSF populations in Figure 7. For instance, at ∼25 fs. There is maximum spin density of C/S on A and minimum CSF population on A, whereas at 30 fs the spin density of C/S on C is minimum and the CSF population on C is maximum (see Figures 8a and 7a, respectively). However, the amplitudes are different. It is clear that the S or Se involvement in the spin density transfer (dashed lines in Figure 8) is much smaller than the allyl C atoms (solid lines in Figure 8).

Data from Figures 7 and 8 lead us to conclude that the difference between (S,S) bisdithiazolyl and (Se,Se) bisdiselenazolyl electron dynamics, in the absence of coupled nuclear motion, must result from the effect of the intervening atoms that act as a superexchange bridge or linker (see Figure 7c,d). The remaining question is whether or not some aspects of the dynamics could be rationalized/predicted from the initial superposition itself. Tables 2 and 3 show the three lowest lying adiabatic states (eigenvalues and corresponding eigenvector with contributions arising from CSF A, B and C, as in Table 1) for a π-stack model of 4 (S,S)/(Se,Se) units, respectively. One can conclude from Tables 2 and 3 that eigenvector *k* = 1 is dominated by CSF B in both cases with only smaller mixing of CSF A and CSF C. The initial superposition of 4 cationic states that results from localizing the hole on fragment A is given in the first column of Tables 2 and 3 in the same format as Table 1 (the contribution of CSF D is negligible and not shown). For (Se,Se) bisdiselenazolyl, the initial superposition involves mainly eigenvectors *k* = 2 and *k* = 3. Here sum of *k* = 2 and *k* = 3 gives mainly CSF C while the difference is CSF A. It thus follows that for (Se,Se) the dynamics is dominated by CSF A and C with an energy difference (*k* = 2 and *k* = 3) of *ca*. 0.05 eV which would in turn give an oscillation period of ca. 80 fs. in reasonable agreement with Figure 7b,d. For (S,S) bisdithiazolyl, this initial superposition involves mainly eigenvector *k* = 2 with smaller contributions from eigenvectors *k* = 1 and *k* = 3, so the 3 state electron dynamics is more complicated.

**Table 2:** Adiabatic States *k* = 1 to 3 Resulting from 4-Unit (S,S) Bisdithiazolyl π-Stack^a^

eigenvector *k*	energy in eV	*A*	*B*	*C*
*k* = 1	0.0000	–0.4	+0.9	+0.3
*k* = 2	0.0932	+0.8	+0.2	+0.5
*k* = 3	0.1326	–0.4	–0.4	+0.7

a Note Cl atoms and ethylene fragment have been replaced by H atoms (format as in Table 1).

**Table 3:** Adiabatic States *k* = 1 to 3 Resulting from 4-Unit (Se,Se) Bisdiselenazolyl π-Stack^a^

eigenvector *k*	energy in eV	*A*	*B*	*C*
*k* = 1	0.0000	+0.3	+0.8	+0.4
*k* = 2	0.1006	–0.6	–0.1	+0.8
*k* = 3	0.1516	+0.7	–0.5	+0.5

a Note Cl atoms and ethylene fragment have been replaced by H atoms (format as in Table 1).

In both cases, the CSF B is mixed with a period ∼35 fs. corresponding to 0.114 eV, which corresponds to roughly the energy difference of the mean of eigenvalues 2 + 3 and eigenvalue 1 (see Figure 7a,b). Thus, the essential difference between bisdithiazolyl and bisdiselenazolyl is the extent to which CSF B is involved. In bisdiselenazolyl, the CSF B acts like a superexchange bridge. In contrast, for bisdithiazolyl, although CSF B still acts as a bridge, it is less strongly coupled; so, the transfer is mainly direct A → C.

**Figure 9:**
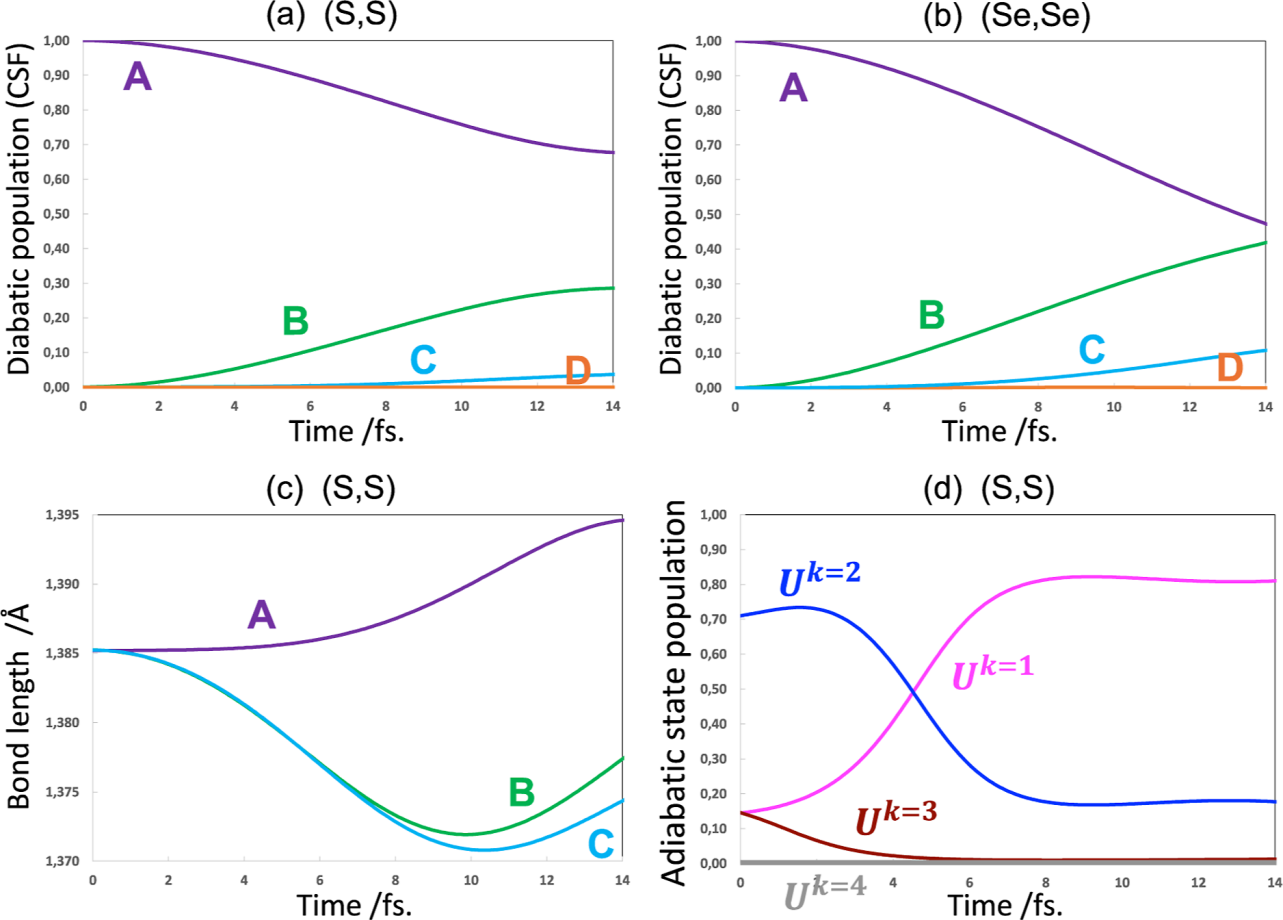
CSF diabatic populations for (a) (S,S) bisdithiazolyl and (b) (Se,Se) bisdiselenazolyl dynamics with coupled nuclear motion. For (S,S): (c) Allyl CC bond lengths, and (d) adiabatic state populations.

We now turn to a discussion of the coupled nuclear motion in the (S,S) bisdithiazolyl or (Se,Se) bisdiselenazolyl radicals. In each case, the coupled nuclear motion observed is an oscillatory nuclear motion mainly involving the allyl carbons on the pyridine radical chromophore. The computation of the gradient is demanding (3N times the cost of the energy where N is the number of atoms). So, we will discuss the results for both (S,S) and (Se,Se) and give a more detailed insight for the (S,S) case (see Figure 9).

The (S,S) and (Se,Se) results are given in Figure 9a,b, respectively. On comparing Figure 9a with 7a and 9b with 7b, one can see that the electron transfer is retarded with the coupled nuclear motion; but qualitatively similar. In particular the enhancement of the role of B as a superexchange bridge for (Se,Se) is qualitatively similar to and without nuclear motion. The allyl bond lengths (Figure 9c) have the expected behavior: *d*_A_ is lengthened as the hole fills with an electron, while *d*_B_ and *d*_C_ are shortened as a result of hole creation. One can also see that there is a transfer of adiabatic population at the first turning point of the nuclear potential energy, around 5 fs in Figure 9d.

For (Se,Se), the experimental conductivity is 3.0 × 10^–4^ S·cm^–1^, while (S,S) exhibits a much smaller experimental conductivity of 3.2 × 10^–6^ S·cm^–1^.^8^ At this point, we are left with a mechanistic explanation. For (Se,Se), we observe a bridge assisted A –[B]→ C transfer (superexchange involving B), whereas for (S,S) the A → C transfer is direct with only small involvement of B (compare Figure 7c,d). In other work,^22^ we have shown that such mechanisms can be seen even in the same molecule with different initial superpositions. Thus, we conclude that, where both electron transfer processes are possible, the long-range superexchange-mediated (involving the linker B) process in (Se,Se) is preferred. Overall, our results point out that superexchange is more efficient than direct exchange in agreement with previous results regarding the study of a model bis(hydrazine) radical cation.^22^

## 3 Conclusions

Real-time CASSCF (Ehrenfest) simulations, with and without nuclear motion, have been carried out as a demonstration for hole and exciton transport in a π-stacked group of: (1) perfectly eclipsed ethylenes, (2) perfectly eclipsed model pyridine radical chromophores, and (3) 4 (S,S) bisdithiazolyl/4 (Se,Se) bisdiselenazolyl radicals. In the “ideal” cases (1) and (2), one observes transfer from the initial molecular unit to the last, where the intermediate units act as a superexchange bridge. The coupled nuclear motion is synergic and the bonds of the first and last units behave asymmetrically (filling a hole with an electron lengthens the bond, while creating a hole shortens it). Interestingly, in the target system of 4 molecular (S,S) or (Se,Se) units, the transfer is between units 1 and 3 (namely, A and C) because of the slippage of the molecular units (equivalent to crystal defects or intermolecular vibrations), i.e., the charge migration cannot propagate across the entire system; instead, it shows coherence length of 3 units. Nonetheless, besides slippage, the target systems are qualitatively similar to the “ideal” model cases. Thus, we have generated a dynamical chemical reaction path driven by the quantum superposition of *n* cationic states (where *n* is the number of molecular fragments in the π-stack model).

The feature that distinguishes the dynamics of (S,S) bisdithiazolyl or (Se,Se) bisdiselenazolyl radical species with 4 molecular units (A–D) is the role played by the bridge. If it is strongly coupled, as in the (Se,Se) bisdiselenazolyl system, there is a superexchange A –[B]→ C charge transfer. Yet, if it is more weakly coupled, as in the (S,S) bisdithiazolyl system, the charge migration is effectively direct: from A to C.

We must stress that the computations, without nuclear motion cost almost nothing since the geometry does not change. Thus, understanding the initial adiabatic state superposition of cationic states and the dynamics without nuclear motion gives considerable insight. The nuclear motion is not strongly coupled (we have documented this elsewhere^27^) so that this physical insight is preserved throughout the electron dynamics.

Finally, let us comment on the fact that our computations are run without any temperature effects. Our simulations would thus be relevant to conductivity in the low-temperature regime. Note that to account for temperature one must go to parametrized models (see reviews^1,2^ for possible strategies). Instead, our approach belongs to the family of direct electronic structure methods,^1^ of which real-time DFT^75^ is an example. Also note that with real-time DFT one needs to use some localization method. Instead, the real-time CASSCF approach used here does depend on a choice of localized orbital or NBO orbitals, which are employed in the selection of the initial conditions (i.e., the choice of fragment CSF).

We believe real-time CASSCF (Ehrenfest) computations may be useful, even if they can only be run for a short time and for a small number of molecular units, because they can be used to understand the atomistic nature of the charge transfer (via the spin density condensed to atoms), the role of structural changes (e.g., slippage which can align with crystal defects or intermolecular vibrations in synthesized molecular materials), and initial length of the polaron. Further, as we have indicated, the bisdithiazolyl and bisdiselenazolyl radical species have different charge carrier mechanisms, namely, direct (A → C) mechanism for (S,S) and superexchange (A –[B]→ C) mechanism for (Se,Se).
